# Safety and long-term efficacy of thoracoscopic Epicardial ablation in patients with paroxysmal atrial fibrillation: a retrospective study

**DOI:** 10.1186/s13019-019-1018-4

**Published:** 2019-11-06

**Authors:** John Johnkoski, Bryan Miles, Anna Sudbury, Mohammed Osman, Muhammad Bilal Munir, Sudarshan Balla, Mina M. Benjamin

**Affiliations:** 1 Department of Cardiothoracic Surgery, Aspirus Wausau Hospital, 2400 Pine Ridge Blvd, Wausau, WI 54401 USA; 2 School of Medicine, Medical College of Wisconsin, 8701 W Watertown Plank Rd, Wauwatosa, WI 53226 USA; 3 Department of Internal Medicine (Division of Cardiovascular Medicine), West Virginia University Hospitals, 1 Medical Center Dr, Morgantown, WV 26506 USA

**Keywords:** Atrial fibrillation, Arrhythmia, Thoracoscopic, Epicardial ablation, Anticoagulation, Pulmonary vein isolation, Implantable loop recorder

## Abstract

**Background:**

The aim of this study is to report the long-term efficacy and safety of thoracoscopic epicardial left atrial ablation (TELA) in patients with paroxysmal atrial fibrillation (AF).

**Methods:**

This was a retrospective review of medical records. We included all patients diagnosed with paroxysmal AF who underwent TELA at our institution between 04/2011 and 06/2017. TELA included pulmonary vein isolation, LA dome lesions and LA appendage exclusion. All (*n* = 55) patients received an implantable loop recorder (ILR), 30 days post-operatively. Antiarrhythmic and anticoagulation therapy were discontinued at 90 and 180 days postoperatively, respectively, if patients were free of AF recurrence. Failure was defined as ≥two minutes of continuous AF, or atrial tachycardia.

**Results:**

Fifty-five patients (78% males, mean age = 61.6 years) qualified for the study. The average duration in AF was 3.64 +/− 3.4 years, mean CHA2DS2-VASc Score was 2.0 +/− 1.6. The procedure was attempted in 57 patients and completed successfully in 55 (96.5%). Two patients experienced a minor pulmonary vein bleed that was managed conservatively. Post procedure, one patient experienced pulmonary edema, another experienced a pneumothorax requiring a chest tube and another experienced acute respiratory distress syndrome resulting in longer hospitalization. Otherwise, there were no major procedural complications. Success rates were 89.1% (*n* = 49/55), 85.5% (*n* = 47/55) and 76.9% (*n* = 40/52) at 6, 12 and 24 months, respectively. In the multivariate cox-proportional hazard model, survival at the mean of covariates was 86 and 74% at 12 and 24 months, respectively.

**Conclusion:**

In this single center experience, TELA was a safe and efficacious procedure for patients with paroxysmal AF.

## Introduction

Atrial fibrillation (AF) is the most common cardiac arrhythmia with several adverse consequences related to a reduction in cardiac output, and to ischemic cerebrovascular accidents or peripheral embolization due to atrial and atrial appendage thrombus formation [1]. Though AF is an independent risk factor of mortality, a causal relationship has not been established [2]. An attempt to maintain sinus rhythm is made in patients based on the presence or absence of symptoms and evidence that myocardial function is being compromised [3]. The most commonly performed invasive procedure used to maintain sinus rhythm is catheter ablation. The efficacy of catheter ablation may vary considerably, depending on the strategy and technology used and the stage of the electroanatomic disease [4]. In 2006, Wolf et al. [5] described a video-assisted thoracoscopic surgical ablation consisting of pulmonary vein isolation (PVI) from the epicardial side with a bipolar radiofrequency clamp, ablation of ganglia over the left atrial (LA) surface, and excision of the LA appendage. This procedure is performed with video-assisted thoracoscopic access to the epicardial space through small, either right-sided or bilateral thoracic incisions and primarily focuses on PVI. The pulmonary veins are electrically isolated with a bipolar radiofrequency ablation clamp or suction assisted unidirectional device. Additional ablation of ganglia or the left atrium can also be performed epicardially. As a stand-alone procedure, thoracoscopic epicardial left atrial ablation (TELA) is limited to PVI, either as a box or islands around each side. An endocardial approach is necessary to connect any lesion to either the mitral or tricuspid valves. Initial efficacy of TELA has been reported to be > 90% in selected populations [6, 7]. Similar to traditional catheter ablation, there is no evidence that survival is improved with TELA [8, 9]. The need for long-term anticoagulation after surgical ablation of AF remains unclear. TELA may reduce stroke risk and therefore the need for long-term anticoagulation. HRS/EHRA/ECAS/APHRS/SOLAECE expert consensus statement on catheter and surgical ablation of atrial fibrillation recommends stand-alone surgical ablation as a reasonable option for persistent and long-standing patients who have failed one or more attempts at catheter ablation who prefer a surgical approach after review of safety and efficacy of options. For paroxysmal AF, it may also be considered after one or more catheter attempts [10]. The Society of Thoracic Surgeons 2017 Clinical Practice Guidelines for the Surgical Treatment of Atrial Fibrillation recommends stand-alone surgical ablation for symptomatic AF without structural heart disease in patients who have failed a class I or III antiarrhythmic medication or catheter-based therapy [11]. The aim of this retrospective study was to assess the long-term efficacy and safety of TELA as a stand-alone procedure in patients with paroxysmal atrial fibrillation.

## Methods

This study was a retrospective review of medical records. We included all patients diagnosed with paroxysmal AF who underwent TELA at our institution between 04/2011 and 06/2017. The patients did not tolerate or have failed antiarrhythmic drugs. At that time interval, there was no dedicated electrophysiology service at the hospital. The surgeon (JJ), explained the TELA procedure to patients. Patients were also informed that catheter-based PVI was the gold standard for ablation procedures, and they were offered a referral to a center where catheter-based ablation can be performed. Patients who denied a referral and opted to undergo TELA were included in this study.

Exclusion criteria for the procedure were previous lung lobectomy, or pericardial surgery and a body mass index above 35 kg/m^2^. Patients’ anticoagulation drugs were held prior to the procedure and resumed the night following the procedure. The procedure included PVI, ganglia mapping and ablation, left atrial dome lesions, superior vena cava lesion, and left atrial appendage exclusion. Under general anesthesia, three 10 mm ports sites were created. The right side was performed first. The pericardium was opened and retracted posteriorly. Using the AtriCure® Transpolar Pen, the autonomic ganglia were mapped and ablated, and then the AtriCure® Lumitip Dissector was used to encircle the pulmonary venous hilum and to guide the AtriCure® Synergy Bipolar RF Clamp around the veins. Three separate lines of ablations were performed with each being repeated twice. Bidirectional block was then confirmed with the pen at each location. An opening was created between the superior vena cava and venous hilum. Working through the transverse sinus, the right-sided portion of the LA dome lesion was created with the AtriCure® Coolrail linear pen. This lesion was repeated twice. Working through the oblique sinus, the right-sided portion of the LA floor lesion was created, and this lesion was repeated twice as well. A superior vena cava lesion is created well above the area of the sinus node with a single application of the radiofrequency clamp. On the left side, the autonomic ganglia and veins were treated identically, and the dome and floor lesions were completed. Bidirectional block was confirmed for the both veins and the box. Autonomic ganglia were localized in the anterior and posterior fat pads, by their response to high-frequency stimulation. Successful ganglion ablation was defined by abolition of the heart rate response (≥50% increase in R-R interval) to high-frequency stimulation at the same regions that had elicited clear responses before ablation. Coolrail® device was then used to perform the LA appendage connecting lesion, which consisted of a line on the posterior surface of the LA appendage and several stamping lesions over the base of the LA appendage in the coumadin ridge area. Finally, the LA appendage was excluded with AtriClip® with the aid of transesophageal imaging to confirm a residual of less than 1 cm at the base. Bidirectional block was then confirmed on the LA appendage. The pericardium was then reapproximated, and a chest tube placed. Chest tubes were typically pulled later the day of the procedure. Patients were discharged from the hospital, the day following the procedure, unless a complication occurred. All patients received an implantable loop recorder (ILR), 30 days post-operatively. Antiarrhythmic and anticoagulation therapy were discontinued at 90 and 180 days postoperatively, respectively, if patients were free of AF recurrence. Failure was defined as 2 min or more, of continuous AF or flutter on any ILR download. Procedural complications were defined as major when prolonging or causing a hospital admission within 30 days.

### Statistical analysis

Continuous data are reported as mean ± standard deviation, and categoric data are reported as number of subjects and ratios. The differences between the groups were compared using the independent-samples Student’s t-test. Chi-square statistics were used to compare categorical variables between groups. Cox-proportional hazard survival analysis was also conducted. Variables were entered in the model if their univariate test *p*-value was ≤0.2. Statistical analysis was conducted using MedCalc Statistical Software version 16.4.3 (MedCalc Software, Ostend, Belgium; https://www.medcalc.org; 2016).

## Results

**Fig. 1:**
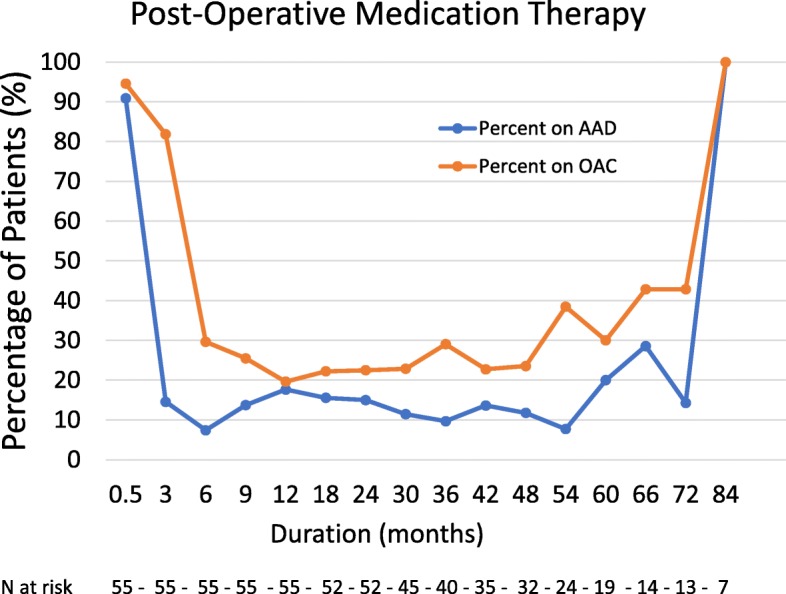
Graphical representation of the percentage of patients on oral anticoagulation (Blue) and antiarrhythmic drugs (Orange) over time (in months), following TELA

**Fig. 2:**
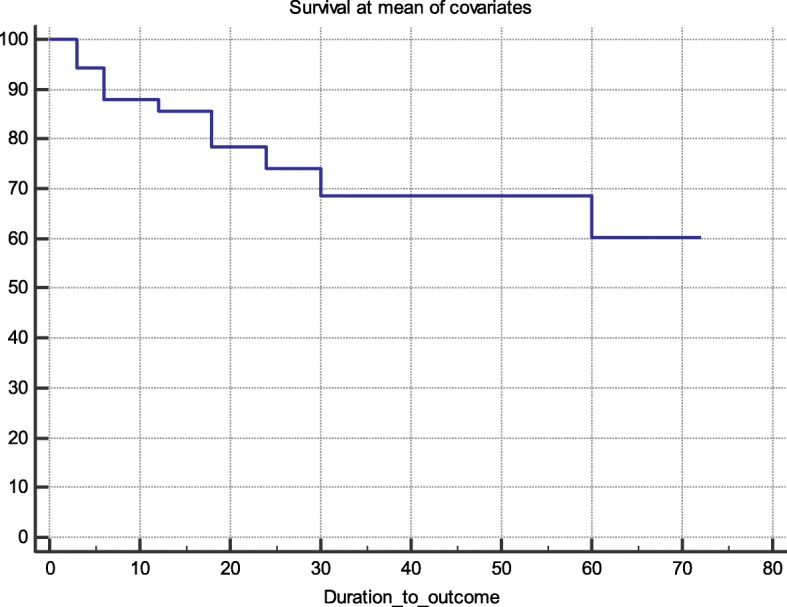
Cox-proportional hazard model of the freedom from atrial arrhythmias over time (in months) following TELA, at the mean of the following covariates: age, hypertension, obstructive sleep apnea, left atrial volume and mitral regurgitation

Fifty-five patients underwent TELA during the study period. Seven (12.5%) had previous catheter ablation procedures before the index procedure. Baseline patient demographics, comorbidities, and echocardiographic characteristics are included in Table 1. The mean age of patients was 61.6 +/− 10.7 years. Forty-four (80%) were men. The mean duration in AF was 3.6 +/− 3.4 years and the mean CHA2DS2-VASc Score was 2.0 +/− 1.6. None of the patients had severe valvular disease. The mean left atrial volume index was 29.3 +/− 8 mL/m^2^. The mean follow-up period was 36.8 +/− 21 months. Figure 1 shows a graphical representation of the percentage of patients receiving oral anticoagulation and/or antiarrhythmic medications at different time points. Success rates were 89.1% (*n* = 49/55), 85.5% (*n* = 47/55) and 76.9% (*n* = 40/52) at 6, 12 and 24 months, respectively. In the multivariate cox-proportional hazard model, survival at the mean of covariates was 88, 86 and 74% at 6, 12 and 24 months, respectively (Fig. 2). None of the patients in our study experienced cerebrovascular accidents during follow up.

**Table 1:** Baseline demographics, clinical characteristics, and echocardiographic values

	All (*n* = 55)	No recurrence (*n* = 36)	Recurrence (*n* = 19)	P-value	HR	95% CI
Demographics						
Age	61.6 +/− 10.7	60.0 +/− 11.6	64.9 +/11.6	0.104	1.02	0.99–1.07
Males	44 (80.0%)	26 (72.2%)	18 (94.7%)	0.722	0.82	0.27–2.48
Duration in AF (years)	3.6 +/− 3.4	3.5 +/− 3.5	3.4 +/− 3.0	0.513	1.35	0.84–1.53
CHA2DS2-VASc Score	2.0 +/− 1.6	1.9 +/− 1.5	2.2 +/− 1.7	0.89	0.98	0.73–1.3
Comorbidities						
Hypertension	32 (58.2%)	17 (47.2%)	15 (78.9%)	0.187	1.69	0.64–4.46
Hyperlipidemia	26 (47.3%)	13 (36.1%)	13 (68.4%)	0.686	1.2	0.49–2.97
Obstructive sleep apnea	5 (9.1%)	2 (5.6%)	3 (15.8%)	0.035	3.34	1.09–10.28
Coronary artery disease	7 (12.7%)	3 (8.3%)	4 (21.1%)	0.745	0.81	0.24–2.81
Cerebrovascular disease	0	0	0			
Diabetes mellitus	6 (10.9%)	4 (11.1%)	2 (10.6%)	0.981	1.02	0.23–4.42
Echocardiographic values						
Ejection Fraction (%)	61.8 +/− 5.6	61.0 +/− 6.2	62.2 +/− 4.3	0.506	1.03	0.94–1.19
Mitral regurgitation					4.01	0.93–20.57
Mild	35 (63.6%)	17 (47.2%)	17 (89.5%)	0.056		
Moderate or severe	0	0	0			
Tricuspid regurgitation					1.81	0.51–6.46
Mild	30 (54.5%)	18 (50.0%)	12 (63.3%)	0.99		
Moderate	1 (1.8%)	0	1 (5.3%)	0.57		
RA dilation					1.52	0.48–4.83
Mild	10 (18.2%)	5 (9.1%)	5 (26.3%)	0.484		
Moderate	2 (3.6%)	0	2 (10.5%)	0.180		
LA diameter (cm)	4.0 +/− 0.6	3.9 +/− 0.6	4.0 +/− 0.5	0.296	1.74	0.79–5.01
LA volume index (cm/m2)	29.3 +/− 8.0	27.4 +/− 5.8	31.1 +/− 9.4	0.374	1.56	0.42–5.93

The procedure was attempted in 57 patients and completed successfully in 55 (96.5%) patients. One patient experienced tension pneumothorax and the other experienced a major bleed during TELA and those two patients were converted to a full sternotomy for AF ablation. Two patients experienced a minor pulmonary vein bleed that was managed conservatively. Post procedure, one patient experienced pulmonary edema, another experienced a pneumothorax requiring a chest tube and another experienced acute respiratory distress syndrome resulting in longer hospitalization. Otherwise, there were no procedural complications, including phrenic nerve injury, esophageal fistula, major bleeding, hemothorax, cardiovascular or cerebrovascular events. At the end of the follow up period, none of the patients experienced any cerebrovascular accidents.

## Discussion

This study investigated the long-term efficacy and safety of TELA as a stand-alone procedure in paroxysmal AF patients, using ILR for follow up. The major finding of our study is that the efficacy of TELA was similar to previously published studies using catheter-based PVI.

n a metanalysis of 19 studies, the pooled 12-month success rate for the 11 studies reporting outcomes for paroxysmal AF patients was 66.6% (95% CI 58.2 to 74.2%). Single-procedure freedom from atrial arrhythmia at long-term follow-up was 54.1% (95% CI 44.4 to 63.4%) in paroxysmal AF patients. The multiple-procedure long-term success in paroxysmal AF was 79.0% in 8 studies (95% CI 67.6 to 87.1%) [9].

The efficacy results of our study are similar to previous TELA studies. There is a limited number of publications which reported the long-term efficacy of TELA as a stand-alone procedure for restoration of sinus rhythm in AF patients, likely because it was first described -only- a little more than a decade ago. Edgerton et al. reported the results of a prospective, nonrandomized study of 52 consecutive patients with symptomatic paroxysmal AF undergoing TELA [6]. Unlike our study where all patients received an ILR, monitoring was obtained by either a 24-h Holter monitor, 2- to 3-week event monitoring, or interrogation of an implanted pacemaker. Freedom from AF/flutter/tachycardia was 86.3 and 80.8% at 6 months and 1 year, respectively. The FAST trial randomly assigned 124 patients with antiarrhythmic drug-refractory AF with LA dilatation and hypertension (33%) or failed prior catheter ablation (67%) to either minimally invasive surgical ablation or catheter ablation. At 12 months, the primary end point of freedom from LA arrhythmia of greater than 30 s without antiarrhythmic drugs was significantly higher in the surgical ablation group (65.6 versus 36.5%). However, there were significantly more periprocedural complications such as pneumothorax, major bleeding, and the need for pacemaker in the surgical ablation group (35.4 versus 15.9%) [8]. Rosati et al., reported the results of biatrial ablation in 49 consecutive patients with AF who underwent right-sided monolateral thoracoscopic ablation. Twenty-four hours ECG Holter monitoring was used as the primary means of defining AF recurrences. At 13 months, 43 patients (87.7%) of patients were in sinus rhythm; 35 (71.4%) were free from antiarrhythmic drugs and 37 (75.5%) from oral anticoagulation [12].

None of the patients in our study experienced cerebrovascular accidents during follow up. With an average CHA2DS2VASc score of 2, the estimated stroke risk for our population would be 2.2% per year and 2.9% combined risk of stroke, transient ischemic attacks or systemic embolization [13, 14]. The novelty of this study comes from exclusively studying paroxysmal AF patients with a longer follow up period than the previous reports. Also, all patients received an ILR, which can identify AF recurrences when they are very infrequent [15].

### Limitations

Limitations of this study include all the inherent limitations of a retrospective study, and the absence of a control group. The Heart Rhythm Society defines failure following an ablation procedure as more than 30 s of AF and this study used a cutoff of 2 min which is the lowest reported by an ILR. Also, there were no postprocedural studies conducted to assess the restoration of atrial mechanical function, in this cohort.

## Conclusions

In this retrospective single center experience, TELA was a safe and efficacious procedure for patients with paroxysmal AF. Long-term outcomes are similar to the gold standard of catheter-based PVI. Results need to be validated in a randomized clinical trial fashion to compare the safety, efficacy and quality of life metrics between patients with atrial fibrillation undergoing thoracoscopic epicardial versus catheter-based ablation procedures.

## Data Availability

All data generated or analysed during this study are included in this published article.

## Abbreviations

AF: Atrial fibrillation
ILR: Implantable loop recorder
PVI: Pulmonary vein isolation
TELA: Thoracoscopic epicardial left atrial ablation
